# Polydatin prevents cholesterol gallstone formation by regulating cholesterol metabolism via PPAR-γ signaling

**DOI:** 10.1515/biol-2022-1009

**Published:** 2025-08-01

**Authors:** Jun Li, Xiaopeng Yu, Zhongxiao Zhou

**Affiliations:** Department of General Surgery, Central Hospital Affiliated to Shenyang Medical College, No. 5 Nanqixi Road, Shenyang, 110024, China; Department of Oncological Surgery, Shengjing Hospital of China Medical University, No. 36 Sanhao Street, Shenyang, 110004, China

**Keywords:** cholesterol gallstone disease, polydatin, inflammation, cholesterol metabolism, PPAR-γ signaling

## Abstract

Polydatin is a stilbene that has been demonstrated to regulate lipid, cholesterol, and glucose metabolism in humans. However, its potential role in cholesterol gallstone formation remains uncertain. C57BL/6 mice were fed a lithogenic diet (LD) and administered polydatin via intragastric administration. At the end of the 8-week study period, the animals were euthanized in order to collect bile/serum samples and gallbladder/liver tissues for subsequent analysis. *In vitro* studies were conducted in which human intrahepatic biliary epithelial cells (HIBECs) were exposed to lipopolysaccharide (LPS) for a period of 24 h. Subsequently, the culture supernatant and cells were harvested for further analysis. The results demonstrated that polydatin markedly reduced cholesterol gallstone formation, attenuated pathological alterations in the gallbladder and liver tissues, and improved lipid profiles in serum and bile samples. Moreover, polydatin exhibited anti-inflammatory properties, regulated cholesterol metabolism-related genes, and activated the PPAR-γ signaling pathway in mice fed an LD diet. In HIBECs, polydatin treatment prevented LPS-induced inflammatory cytokine release, dysregulation of cholesterol metabolism-related genes, and inactivation of the PPAR-γ pathway. This study is the first to demonstrate that polydatin prevents cholesterol gallstone formation by regulating cholesterol metabolism via the PPAR-γ signaling pathway.

## Graphical abstract

**Figure:**
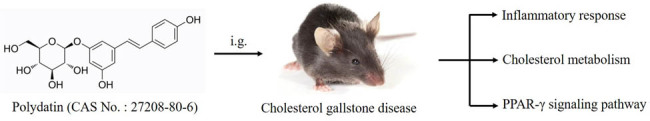

## 1 Introduction

Gallstone disease is a prevalent digestive disorder that gives rise to significant economic concerns [1]. A number of risk factors have been identified as potential contributors to the formation of gallstones. These include age, gender, obesity, diabetes mellitus, dietary habits, and the presence of liver diseases [2,3]. Gallstones can be classified into three main categories: cholesterol gallstones, pigment gallstones, and mixed types [4]. The majority of cases, approximately 80%, are attributed to cholesterol gallstone disease [5]. Currently, cholecystectomy remains the most effective therapeutic strategy, but it is associated with significant risks [6]. Therefore, it is of great importance to seek safer and more effective pharmacological therapies.

Natural products are defined as monomers and mixtures derived from natural sources, including microorganisms, plants, and animals [7]. Extensive research has been conducted on their potential as protective agents for a range of diseases [8,9,10,11]. Polydatin is a stilbene that is primarily isolated from *Polygonum cuspidatum* [12]. It has been demonstrated that this compound possesses anti-cancer, anti-inflammatory, anti-angiogenic, anti-diabetic, and anti-oxidative activities in the context of human diseases [13]. Recently, it has also been demonstrated that polydatin plays a role in lipid and cholesterol metabolism [14]. In mice fed a high-fat diet, polydatin has been shown to inhibit adipose tissue inflammation and improve lipid metabolism [15]. In another study, polydatin has been demonstrated to mitigate atherosclerosis *in vivo* by regulating autophagy [16]. However, its role in cholesterol gallstone formation remains unclear.

In this study, a cholesterol gallstone animal model was generated by feeding a lithogenic diet (LD) to C57BL/6 mice. The aim of the present study was to evaluate the role of polydatin in cholesterol gallstone formation, employing both *in vitro* and *in vivo* approaches.

## 2 Materials and methods

### 2.1 Animals

Male C57BL/6 mice (8 weeks of age; Liaoning Changsheng, China) were randomly assigned to one of three groups (*n* = 6 mice per group). The animals were fed an LD diet containing 0.5% cholic acid, 15% fat, and 1.25% cholesterol for 8 weeks, thereby generating an animal model of cholesterol gallstones, as previously reported [17]. Concurrently, the animals were administered polydatin (50 mg/kg; MedChemExpress, USA) or vehicle (0.5% CMC-Na; MedChemExpress) via intragastric administration once daily for 8 weeks. The dosage and route of polydatin administration were based on a previously published reference [18]. The control mice were provided with standard chow. The mice were euthanized by sodium pentobarbital overdose at the conclusion of the experiment. The samples of bile and serum were collected, and the gallbladders and livers were excised.


**Ethical approval:** The research related to animal use has been complied with all the relevant national regulations and institutional policies for the care and use of animals and has been approved by the Institutional Ethics Committee of Shengjing Hospital of China Medical University (2017PS203K).

### 2.2 Cell treatment

Human intrahepatic biliary epithelial cells (HIBECs; Procell, China) were cultured at 37°C in Dulbecco’s modified Eagle’s medium (DMEM) and 10% fetal bovine serum (FBS; Invitrogen). The cells were exposed to lipopolysaccharide (LPS) (100 μg/mL; Sigma, USA) and polydatin (20 μM) for a period of 24 h prior to harvesting the cells and the culture supernatant.

### 2.3 HE staining

The gallbladder and liver tissues were embedded in paraffin and then sectioned at a thickness of 4 μm. The sections were deparaffinized in xylene and rehydrated in graded ethyl alcohol. Subsequently, the sections were subjected to a 5 min staining process, followed by a 3 s immersion in an alcohol/hydrochloric acid solution (Sinopharm, China) and a subsequent 3 min staining process with eosin (Sinopharm, China). Subsequently, the sections were dehydrated in graded ethyl alcohol, cleared in xylene, and mounted in neutral balsam. The pathological changes were observed using a microscope (Olympus, Japan).

### 2.4 Oil Red O staining

The liver tissues were excised, frozen, and subsequently sectioned. Subsequently, the sections were stained for 5 min with Oil Red O (Solarbio), counterstained with hematoxylin (Solarbio), and washed with distilled water. The images were captured using a microscope (Olympus).

### 2.5 Biochemical and ELISA assays

The total cholesterol (TC) levels in serum and bile were determined using a TC assay kit (Nanjing Jiancheng, China) in accordance with the manufacturer’s instructions. The levels of triglyceride (TG) were determined using a TG assay kit in accordance with the manufacturer’s instructions. The total bile acid levels were quantified using the Total bile acid assay kit (Nanjing Jiancheng) in accordance with the manufacturer’s instructions. The levels of low-density lipoprotein cholesterol (LDL-C) and high-density lipoprotein cholesterol (HDL-C) were determined using the LDL-C and HDL-C assay kits (Nanjing Jiancheng), respectively, in accordance with the manufacturer’s instructions. The levels of pro-inflammatory cytokines, including interleukin 6 (IL-6), tumor necrosis factor-α (TNF-α), and interleukin-1β (IL-1β), in serum and the culture supernatant were examined using Mouse IL-6/TNF-α/IL-1β ELISA Kits (Cloud-Clone Corp., China) in accordance with the manufacturer’s instructions.

### 2.6 Real-time PCR

Total RNA was extracted from liver tissues and HIBECs using the RNAiso Plus lysis buffer (TaKaRa) in accordance with the manufacturer’s instructions. The concentration of RNA was subsequently determined. Reverse transcription was performed using the PrimeScript RT reagent kit with gDNA Eraser (TaKaRa). Real-time PCR was conducted on a CFX96 Real-time PCR System (Bio-Rad Laboratories, USA) using TB Green *Premix Ex Taq* II (TaKaRa). The 2^−ΔΔ*C*^_t_ method was employed for the analysis of relative gene expression. The primer sequences are presented in Table 1.

**Table 1:** Primers for real-time PCR analysis

Genes	Forward primer (5′–3′)	Reverse primer (5′–3′)
mmu-ABCG5	ATTGTCACCATCCACCAG	GACAGGGGTAACCACAGT
hsa-ABCG5	TCTCTTGGCCCCCCACTT	CTATATTTGGATTTTGGACGATACCA
mmu-ABCG8	CACCCTTGTCCTCGCTAT	TCCTTTGCCTCAGCTTTC
hsa-ABCG8	GACAGCTTCACAGCCCACAA	GCCTGAAGATGTCAGAGCGA
mmu-CYP7A1	ATGGAGAAGGCTAAGACG	CACTTCTTCAGAGGCTGC
hsa-CYP7A1	ATCTGGAGAAGGCCAAGACA	TTTCATTGCTTCTGGGTTCC
mmu-MUC5AC	CTGTGACATTATCCCATAAGCCC	AAGGGGTATAGCTGGCCTGA
hsa-MUC5AC	GCTTCCTGCTCCGAGATGT	AAGACGCAGCCCTCATAGAA
mmu-GAPDH	CGTGTTCCTACCCCCAATG	ATGTCATCATACTTGGCAGGTT
hsa-GAPDH	ACACCCACTCCTCCACCTTT	TGACAAAGTGGTCGTTGAGG

### 2.7 Western blotting

Liver tissues and HIBECs were fully ground in radioimmunoprecipitation assay buffer (Beyotime, Shanghai, China) and then lysed for 30 min on ice. Subsequently, the lysates were subjected to centrifugation, after which the resulting supernatant was collected to determine its protein concentration through the utilization of the BCA protein assay kit (Beyotime). The proteins in the lysates were separated by sodium dodecyl sulfate–polyacrylamide gel electrophoresis and transferred to polyvinylidene fluoride membranes from Millipore. The membranes were incubated with primary antibodies against ABCG5 (1:500; 27722-1-AP; Proteintech group, USA), ABCG8 (1:1,000; ab223056; Abcam, USA), CYP7A1 (1:1,000; bs-21429R; Bioss, China), MUC5AC (1:1,000; ab3649; Abcam), or PPAR-γ (1:1,000; ab45036; Abcam) at 4°C overnight. Subsequently, the membranes were incubated with secondary antibodies (1:5,000; ab205718 and ab205719; Abcam) for 45 min. The protein bands were visualized using an electrochemiluminescence reagent (Beyotime), and the band intensity was analyzed using ImageJ software (NIH).

### 2.8 Immunofluorescence

The slides were fixed in 4% paraformaldehyde, treated with Triton X-100, and blocked. Subsequently, the slides were incubated with a PPAR-γ antibody (1:200; Cat No. ab45036; Abcam) at 4°C overnight, followed by an Alexa Fluor 555-labeled secondary antibody (1:200; Cat No. A27039; Invitrogen, USA) for 1 h at 37°C. The nuclei were counterstained with 4′,6-diamidino-2-phenylindole (DAPI) and imaged using an Olympus microscope.

### 2.9 Statistical analysis

The data are presented as mean ± SD. A one-way analysis of variance with a Tukey *post hoc* test was employed to analyze differences when comparing three or more groups. The Student’s *t*-test was employed to analyze the differences between the two groups. A *P*-value of less than 0.05 was considered to be statistically significant.

## 3 Results

### 3.1 Polydatin inhibits cholesterol gallstone formation and alleviates pathological changes in mice

**Figure 1:**
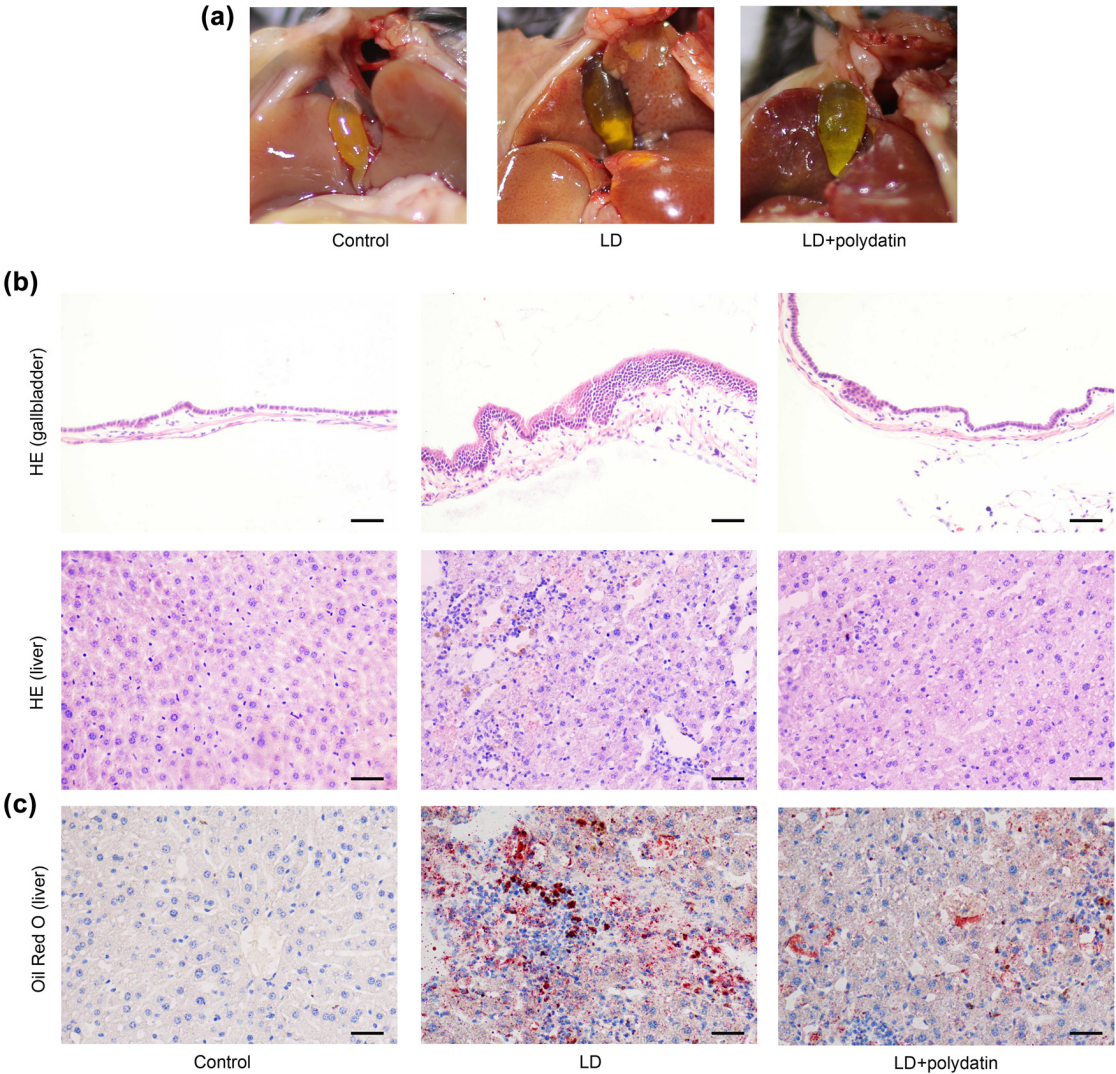
Polydatin inhibits cholesterol gallstone formation and alleviates pathological changes in mice. C57BL/6 mice were fed a lithogenic diet (LD) and intragastrically administered polydatin for 8 weeks. (a) The mice were euthanatized to expose gallbladders. Representative images of gallbladders are shown. (b) Pathological changes in the gallbladder and liver tissues were examined by HE staining. Scale bars represent 50 μm. (c) Lipid deposition in liver tissues was evaluated by Oil Red O staining. Scale bars represent 50 μm.

Following the completion of the animal experiments, the mice were euthanized, and their gallbladders were photographed (Figure 1a). Subsequently, pathological alterations in the gallbladder and liver tissues were investigated by the utilization of HE staining. The administration of an LD diet resulted in the development of several pathological alterations within the gallbladder tissues, including an increase in the thickness of the gallbladder wall. Additionally, the administration of an LD diet resulted in the infiltration of inflammatory cells, the formation of lipid droplets, and a loosening of the arrangement of hepatocytes in the liver tissues. The administration of polydatin was observed to significantly attenuate the aforementioned pathological changes in the gallbladder and liver tissues (Figure 1b). Additionally, no lipid droplets were observed in the livers of the control mice, whereas administration of an LD diet resulted in hepatic lipid accumulation in mice. The administration of polydatin was observed to significantly inhibit the deposition of lipids in liver tissues (Figure 1c).

### 3.2 Polydatin administration improves lipids and bile acids and inhibits inflammation in mice

**Figure 2:**
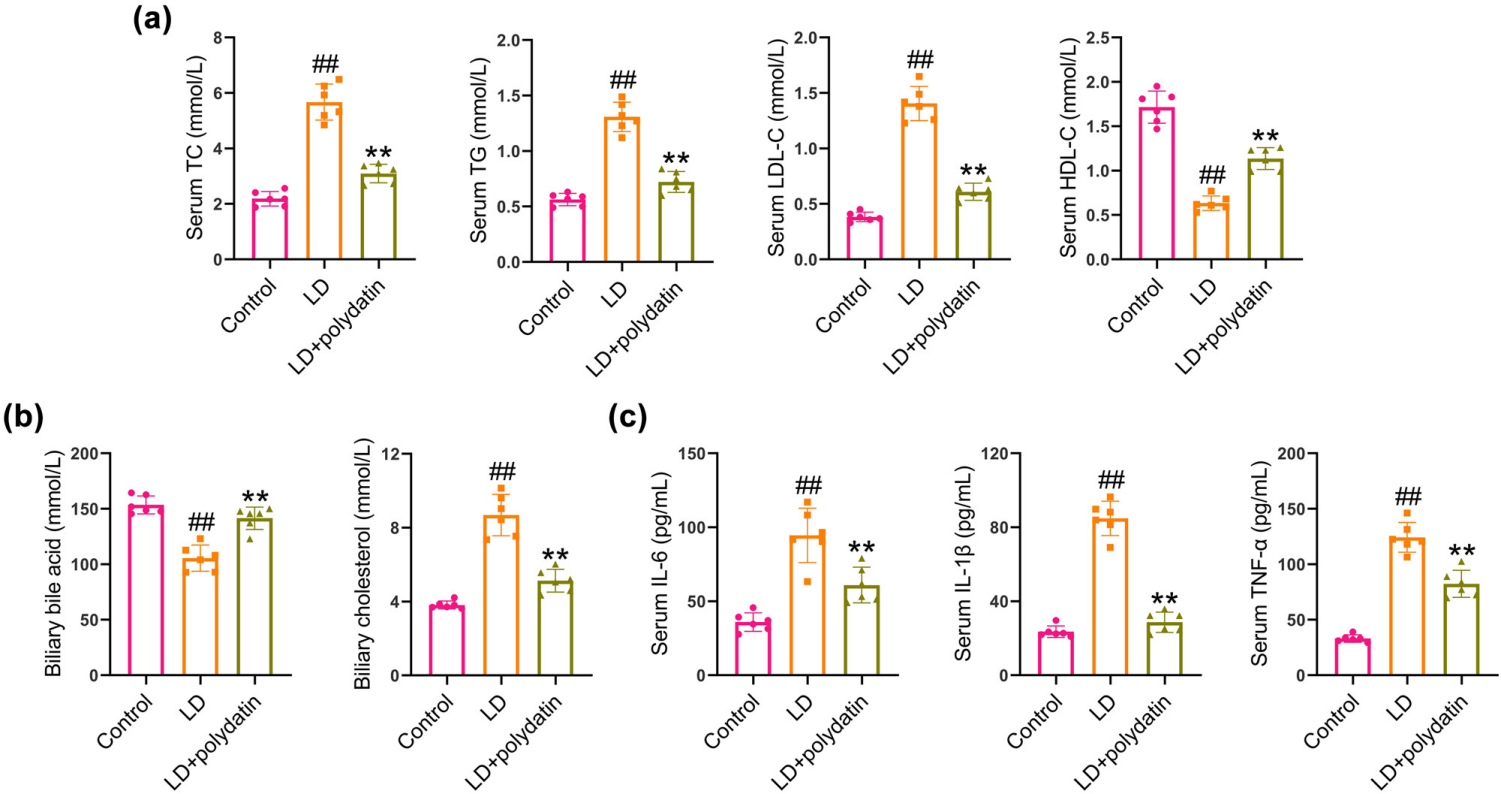
Polydatin regulates lipids and bile acids and inhibits inflammatory cytokine release in mice. (a) After animal experiments, the mice were euthanatized to obtain the serum. Serum levels of HDL-C, LDL-C, TG, and TC were determined. (b) Cholesterol and total bile acid levels were measured in bile samples. (c) Serum levels of pro-inflammatory cytokines were examined by ELISA kits. ^##^ *P* < 0.01 vs control group. **P < 0.01 vs LD group.

Following 8 weeks of dietary intervention involving the LD diet, there was a notable increase in serum levels of LDL-C, TG, and TC, while the level of HDL-C exhibited a decline. The administration of polydatin was observed to significantly decrease LDL-C, TG, and TC levels while simultaneously increasing the HDL-C level in serum (Figure 2a). Additionally, LD-fed mice demonstrated a reduction in total bile acid and an elevation in cholesterol levels in bile samples in comparison to control mice. The administration of polydatin resulted in an increase in total bile acid levels and a reduction in cholesterol levels in bile samples (Figure 2b). The impact of polydatin on the inflammatory process associated with cholesterol gallstone formation was investigated. The LD diet was found to significantly elevate the levels of IL-6, TNF-α, and IL-1β in comparison to the control group. The administration of polydatin resulted in a reduction of these levels in comparison to the untreated model mice (Figure 2c).

### 3.3 Polydatin administration regulates cholesterol metabolism-associated genes and activates PPAR-γ signaling in mice

**Figure 3:**
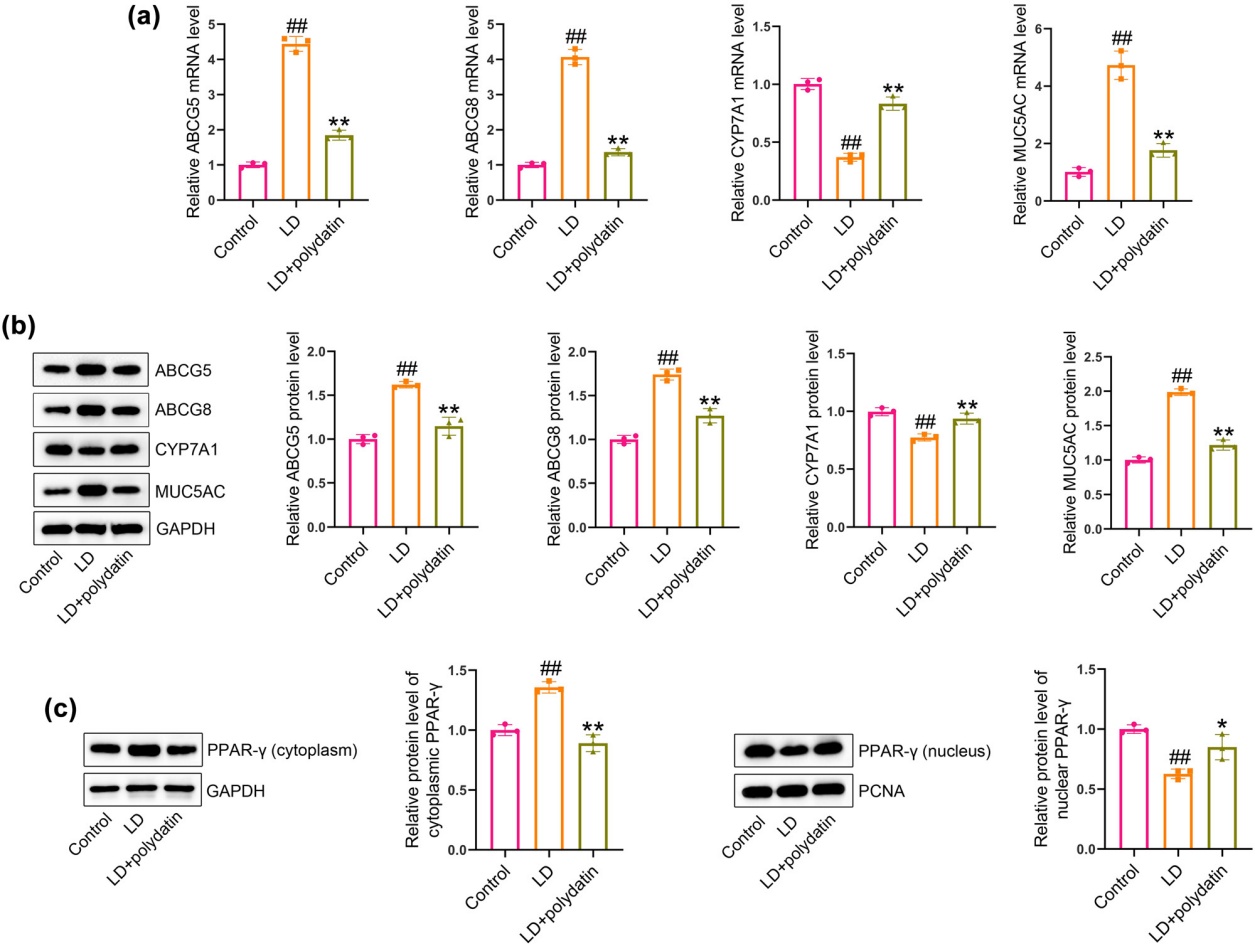
Polydatin regulates cholesterol metabolism-associated genes and activates PPAR-γ signaling in mice. (a) ABCG5, ABCG8, CYP7A1, and MUC5AC levels in liver tissues were detected by real-time PCR. (b) ABCG5, ABCG8, CYP7A1, and MUC5AC protein levels in liver tissues were determined by western blotting. (c) The levels of PPAR-γ in the cytoplasm and nucleus of liver tissues were measured by western blotting. ^##^ *P* < 0.01 vs control group. **P < 0.01 vs LD group.

The mRNA levels of ABCG5, ABCG8, and MUC5AC were found to be significantly elevated in the livers of LD-fed mice, while CYP7A1 mRNA levels were observed to be decreased in comparison to control mice. However, administration of polydatin was observed to significantly decrease ABCG5, ABCG8, and MUC5AC mRNA levels while increasing CYP7A1 mRNA levels in mice when compared to the untreated model mice (Figure 3a). The alterations in these regulatory proteins were validated by western blotting (Figure 3b). Furthermore, the LD diet resulted in a notable increase in cytoplasmic PPAR-γ and a concomitant decrease in nuclear PPAR-γ when compared to the control mice. The administration of polydatin resulted in a reduction in cytoplasmic PPAR-γ and an increase in nuclear PPAR-γ in liver tissues compared to the untreated model mice (Figure 3c).

### 3.4 Polydatin inhibits inflammation in LPS-exposed HIBECs

**Figure 4:**
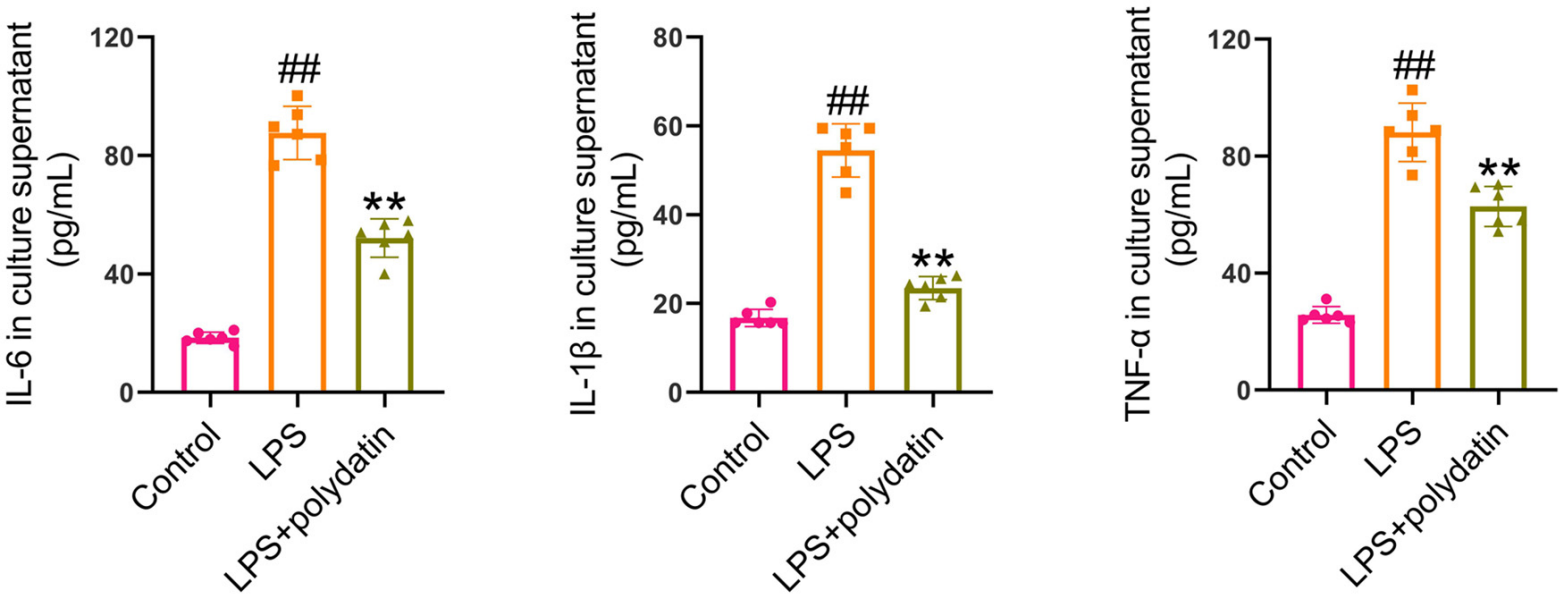
Polydatin reduces inflammation in LPS-exposed HIBECs. HIBECs were exposed to 100 μg/mL LPS and polydatin (20 μM) for 24 h. After treatment, the culture supernatant was harvested. IL-6, IL-1β, and TNF-α levels in the culture supernatant were detected by the ELISA method. ^##^ *P* < 0.01 vs control group. **P < 0.01 vs LPS group.

The anti-inflammatory effects of polydatin were confirmed in intrahepatic biliary epithelial cells exposed to LPS. LPS treatment resulted in a notable elevation in IL-6, TNF-α, and IL-1β levels in the culture supernatant when compared to the control cells. Polydatin treatment resulted in a notable reduction in these levels in comparison to the LPS-exposed cells (Figure 4).

### 3.5 Polydatin regulates cholesterol metabolism and activates PPAR-γ signaling in LPS-exposed HIBECs

**Figure 5:**
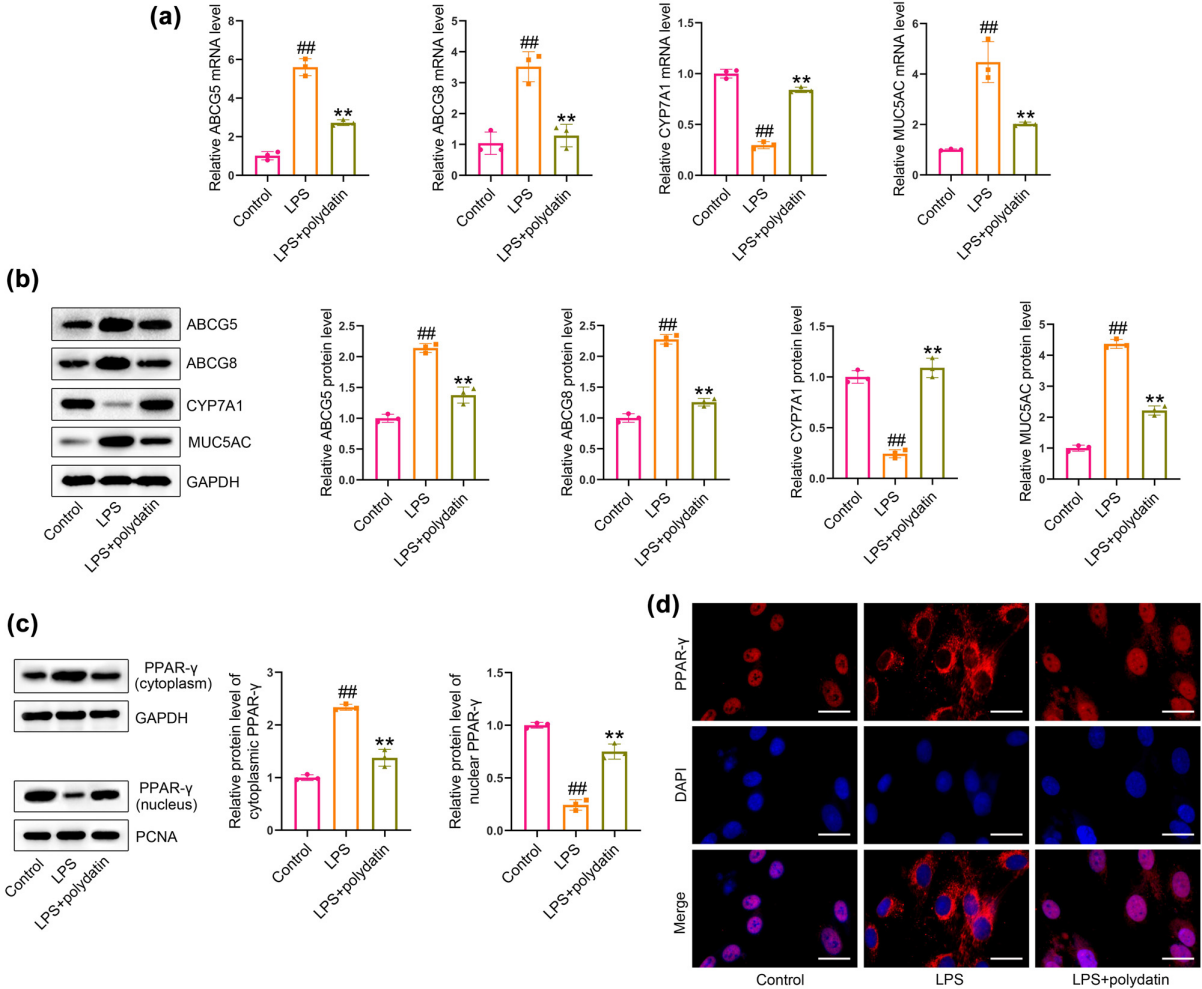
Polydatin modulates cholesterol metabolism-related genes and activates PPAR-γ signaling in LPS-exposed HIBECs. (a) ABCG5, ABCG8, CYP7A1, and MUC5AC levels were measured by real-time PCR. (b) ABCG5, ABCG8, CYP7A1, and MUC5AC protein levels were detected by western blotting. (c) Cytoplasmic and nuclear levels of PPAR-γ were determined by western blotting. (d) Translocation of PPAR-γ was detected by immunofluorescence assay. Scale bars represent 25 μm. ^##^ *P* < 0.01 vs control group. **P < 0.01 vs LPS group.

LPS exposure resulted in a notable elevation in mRNA and protein levels of ABCG5, ABCG8, and MUC5AC, accompanied by a reduction in CYP7A1 levels in HIBECs relative to the control cells. Incubation with polydatin resulted in a notable reduction in the levels of ABCG5, ABCG8, and MUC5AC, while concurrently leading to an increase in CYP7A1 in LPS-exposed HIBECs (Figure 5a and b). Moreover, incubation with LPS resulted in a notable increase in cytoplasmic PPAR-γ and a concomitant decrease in nuclear PPAR-γ when compared to the control cells. Following polydatin treatment, there was a reduction in cytoplasmic PPAR-γ levels and an enhancement in nuclear PPAR-γ levels in LPS-exposed HIBECs (Figure 5c). Furthermore, the immunofluorescence assay provided further evidence of polydatin-induced reactivation of PPAR-γ signaling (Figure 5d).

## 4 Discussion

The formation of cholesterol gallstones is a complex process that is influenced by a number of environmental and genetic factors [19]. A mouse model of LD-induced cholesterol gallstone disease is frequently employed to examine the etiology of cholesterol gallstone formation [20,21]. Currently, there is considerable evidence to suggest that natural products can be effective in the treatment of a number of diseases, including cholesterol gallstone disease [10,11,22,23,24]. The present study demonstrated that polydatin reduced the formation of gallstones, attenuated pathological changes in the gallbladder and liver, regulated cholesterol and lipid metabolism, and inhibited inflammation in C57BL/6 mice fed an LD diet. Furthermore, polydatin was observed to activate the PPAR-γ signaling pathway in liver tissues. The role and potential mechanism of polydatin were validated in LPS-exposed HIBECs.

It has been demonstrated that inflammatory processes are associated with the pathogenesis of a number of diseases, including cancer, ulcerative colitis, doxorubicin-induced renal damage, and septic cardiomyopathy [25,26,27,28,29,30]. Moreover, the formation of cholesterol gallstones is also subject to the influence of inflammatory processes [31,32]. Gallbladder inflammation represents a pivotal element in the pathogenesis of cholesterol gallstones [33]. It has been demonstrated that inflammatory processes regulate hepatic lipid metabolism and biliary cholesterol secretion, which in turn contribute to the formation of cholesterol gallstones [33]. The results demonstrated that pro-inflammatory cytokine levels were elevated in mice fed the LD diet, which is consistent with the findings of a previous study [34]. It is noteworthy that (–)-epigallocatechin-3-gallate has been observed to impede the formation of cholesterol gallstones in mice by curbing the inflammatory response [35]. Furthermore, mice lacking HIF-1α have demonstrated a reduction in both inflammation and mucin deposition within the gallbladder, which has led to a notable decline in cholesterol gallstone formation [36]. Consequently, the inhibition of inflammation may represent a promising avenue for therapeutic intervention in the treatment of cholesterol gallstone disease. The anti-inflammatory activity of polydatin has been demonstrated to attenuate disease progression in a number of contexts, including ulcerative colitis and gouty arthritis [37,38,39]. Polydatin has been demonstrated to alleviate ulcerative colitis in murine models via its anti-inflammatory and antioxidant effects [37]. Additionally, polydatin has been shown to alleviate inflammation and oxidative stress associated with MSU-induced gouty arthritis in mice by regulating PPAR-γ and ferritin activation [38]. Moreover, polydatin has been shown to mitigate hepatic ischemia-reperfusion injury by inhibiting the release of pro-inflammatory cytokines through the modulation of macrophage polarization [39]. Furthermore, polydatin has been shown to alleviate renal damage caused by calcium oxalate crystals through its anti-inflammatory effects [40]. However, further investigation is required to elucidate the role of polydatin in cholesterol gallstone disease. In this study, polydatin was observed to suppress the inflammatory response in C57BL/6 mice fed an LD diet. Subsequently, the role of polydatin was confirmed in an *in vitro* model. In HIBECs, polydatin exhibited partial efficacy in rescuing the release of pro-inflammatory cytokines induced by LPS *in vitro*. The results indicate that polydatin may exert an inhibitory effect on cholesterol gallstone formation by modulating the inflammatory response.

ABCG8 and ABCG5 are ATP-binding cassette transporters that facilitate the excretion of cholesterol from the intestine and liver, thereby regulating cholesterol homeostasis [41]. The presence of ABCG8 and ABCG5 has been demonstrated to correlate positively with an increased risk of developing cholesterol gallstone disease [42]. Moreover, evidence from mouse models indicates that miR-223 knockout increases cholesterol gallstone formation by regulating the expression of ABCG5 and ABCG8 [43]. CYP7A1 is a rate-limiting enzyme that plays a pivotal role in bile acid synthesis in gallstone disease [44]. As reported by Chen et al., the inhibition of PCSK9 has been demonstrated to inhibit cholesterol gallstone formation through the upregulation of CYP7A1 expression [45]. Activation of the constitutive androstane receptor has been demonstrated to contribute to the prevention of cholesterol gallstone formation by decreasing the expression of ABCG5/ABCG8 and increasing that of CYP7A1 [46]. The modulation of these regulators by pharmacological therapy may represent an additional mechanism for the prevention of cholesterol gallstone formation. We found that the administration of polydatin significantly reduced the levels of ABCG5 and ABCG8 in liver tissues while elevating the levels of CYP7A1. Moreover, in LPS-incubated HIBECs, polydatin treatment resulted in a reduction in ABCG5 and ABCG8 levels and an increase in CYP7A1 levels. MUC5AC is one of the gel-forming mucins that is expressed in epithelial cells [5]. The dysregulation of this process has been associated with cholesterol gallstone formation [47,48]. Prior research has demonstrated that MUC5AC expression is increased in individuals with cholesterol gallstone disease [48]. The present study demonstrated that polydatin significantly decreased MUC5AC levels in liver tissues of mice fed an LD diet and exposed to LPS-treated human intestinal epithelial cells. The findings indicate that polydatin may impede cholesterol gallstone formation by modulating cholesterol metabolism.

PPAR-γ is a member of the nuclear hormone receptor superfamily and functions as a ligand-activated transcription factor [49]. The overexpression of PPAR-γ has been demonstrated to result in a reduction of cholesterol levels in normal hepatocytes [50]. Additionally, a separate study has indicated that pioglitazone can prevent cholesterol gallstone formation by upregulating the expression of PPAR-γ [51]. Pravastatin has been demonstrated to inhibit cholesterol gallstone formation in hamsters by activating the PPAR-γ signaling pathway in liver and gallbladder tissues [52]. Moreover, Ruan et al. have demonstrated that polydatin exerts a neuroprotective effect against cerebral injury following ischemic stroke by modulating the PPAR-γ signaling pathway [53]. Nevertheless, further investigation is required to ascertain whether the PPAR-γ signaling pathway is involved in the protective effects of polydatin against cholesterol gallstone formation. The results demonstrated that feeding an LD diet resulted in the inhibition of the PPAR-γ signaling in liver tissues, which was subsequently reactivated by polydatin administration. Furthermore, polydatin was observed to activate the PPAR-γ signaling in HIBECs exposed to LPS. These findings suggest that polydatin may exert an inhibitory effect on cholesterol gallstone formation by activating the PPAR-γ pathway.

## 5 Conclusions

The present study demonstrated that polydatin reduced cholesterol gallstone formation via the inhibition of inflammatory processes and the regulation of cholesterol metabolism, which was achieved by activating the PPAR-γ signaling pathway. Polydatin may be a promising therapeutic agent for the treatment of cholesterol gallstone disease. It is important to acknowledge that the current study was conducted using male mice. To gain a more comprehensive understanding of the effects of polydatin on cholesterol gallstone disease and the underlying regulatory mechanisms, future studies should be conducted in female mice. Furthermore, in future studies, we will use a positive control drug to compare the effect of polydatin on cholesterol gallstone disease, which will provide valuable insight into its potential clinical applications.
